# Whispering Gallery Mode Resonator Temperature Compensation and Refractive Index Sensing in Glucose Droplets

**DOI:** 10.3390/s21217184

**Published:** 2021-10-29

**Authors:** Inga Brice, Karlis Grundsteins, Kristians Draguns, Aigars Atvars, Janis Alnis

**Affiliations:** Institute of Atomic Physics and Spectroscopy, University of Latvia, Jelgavas Str. 3, LV-1004 Riga, Latvia; karlis.grundsteins@lu.lv (K.G.); kristians.draguns@lu.lv (K.D.); aigars.atvars@lu.lv (A.A.); janis.alnis@lu.lv (J.A.)

**Keywords:** PMMA coating, refractive index sensing, resonance scan method, silica microsphere, temperature scanning, whispering gallery mode resonator (WGMR)

## Abstract

Among the different types of photonic sensor devices, optical whispering gallery mode resonators (WGMRs) have attracted interest due to their high level of sensitivity, small size, and ability to perform real-time temperature measurements. Here we demonstrate the applicability of temperature measurements using WGMR in both air and liquid environments. We also show that WGMR allowed measurements of the refractive index variations in an evaporating glucose–water solution droplet. The thermal tuning of WGMR can be reduced by coating WGMRs with a thin layer of polymethyl methacrylate (PMMA). Dip-coating the silica microsphere multiple times significantly reduced the resonance shift, partially compensating for the positive thermo-optical coefficient of silica. The shift direction changed the sign eventually.

## 1. Introduction

Whispering gallery mode resonators (WGMRs) have attracted significant interest because of their extreme sensitivity, compactness, fast responses, and real-time measurements. WGMRs have been used in various applications, such as temperature sensors [1,2,3,4], pressure and force sensors [3], humidity sensors [5,6], accelerometers [7], and biosensors [8,9,10]. WGMRs show ultra-high quality (Q) factors and ultra-narrow linewidth resonances that allow achieving very high resolution and detecting extremely small incremental changes of parameters [11].

WGMR sensors’ operational principles are based on a shift of the resonance frequencies or resonance width due to external influences. A common method for recording the WGMR resonance spectrum is the laser frequency scan method. A slow frequency scan of the laser is finely done by sweeping through the resonances, and the transmission signal is recorded. When the laser frequency matches the resonant frequency of the WGMR, an absorption dip in the transmission spectra is observed [12]. The width of the dip indicates the Q factor of the WGMR.

The Q factor of a WGMR describes the ability to store energy inside. It can be mathematically described as the ratio of the resonant frequency to the full width at half maximum of the resonance. To enhance the light–matter interaction inside the resonator, it is necessary to have a high Q factor. Several losses limit the maximum value of the Q factor. Material losses are caused by the absorption in the material. Silica has low material absorption in visible and telecommunication regions but absorbs water from the moisture of the ambient air limiting the Q below 10${}^{9}$ [13]. Surface scattering losses are provoked by surface roughness and tiny particles, for example, dust or coatings [10]. Radiative losses increase for smaller resonators because of the curved surface, and for resonators in liquid environments because the refractive index contrasts between the resonator material and media are reduced. These losses are defined by the resonator cavity and the surrounding medium. Coupling losses, however, can be adjusted by controlling the position and distance between the resonator and the coupling element [14].

WGMRs are very sensitive to the changes in temperature due to the thermo-optical and thermal expansion coefficients of the resonator material. The thermo-optical coefficients for different materials can be either positive (silica) or negative (CaF${}_{2}$, PMMA, PDMS), and they determine the shift directions of the resonances. WGMR sensitivity to temperature variations could be potentially both useful [15] and troublesome [10] for applications in different photonic devices. The thermal effects can influence negatively, for example, the generation of Kerr comb [16,17]. Silica is very sensitive to temperature changes [1,4,18]. Several attempts have been made to reduce the thermal sensitivity of WGMRs—for instance, controlling the resonator size and optical properties of the medium [19] or coating it by a thin layer of material with opposite thermo-optical properties [20,21,22,23]. By adjusting the layer thickness, the thermal sensitivity of the resonator can be compensated. The layer can be applied using a method based on the chosen material and WGMR geometry: dip-coating [23], drop coating [22], sputtering [20], etc.

In this paper, temperature and refractive index sensing with whispering gallery mode resonances in air, water, and glucose droplet media are reported. A Peltier element was used to control the temperature of the measurement. The change in refractive index of the evaporating liquid droplet was observed during the thermal tuning of the WGMR. The application of the proposed method for chemical sensing is discussed. To reduce temperature sensitivity, silica microspheres were coated with a thin layer of material, polymethyl methacrylate (PMMA).

## 2. Materials and Methods

### 2.1. Fabrication of Silica Microspheres

**Figure 1:**
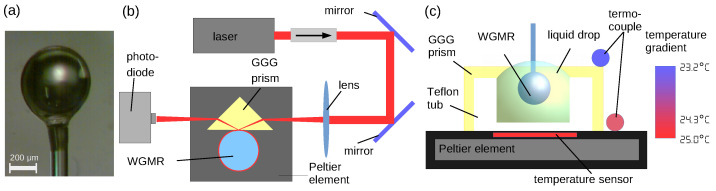
The scheme of the experimental system. (**a**) Silica microsphere whispering gallery mode resonator (WGMR) with a diameter of (509 ± 3) μm. (**b**) View from the top with a Gadolinium Gallium Garnet (GGG) coupling prism to excite the resonance of whispering gallery mode resonator (WGMR) using a 780 nm external cavity diode laser (ECDL), a Peltier element to control temperature, and a photodiode to process the signal transmitted in the air. (**c**) Side view where a Teflon tub is viable to contain the liquid drop, which can be situated above the rim because of liquid tension forces.

WGMRs were fabricated from a standard Corning SMF28 Optical Telecommunication fiber. A hydrogen–oxygen flame torch was used as a heating source. The fiber jacket was stripped. The protective coating was removed using an acetone bath and then cleaned using ethanol to wipe the dust particles from the surface. The striped and cleaned telecom fiber was fastened to a step motor which moved the fiber towards the flame with a constant speed. As the silica melted, a microsphere grew on the tip of the fiber stem due to surface tension. To achieve higher Q factors, the fabricated sphere was moved to the cooler part of the flame for 10–20 s. The results of the fabrication process were WGMR spheres with diameters of 300–700 μm and Q factors of up to $10^{8}$ (see Figure 1a).

To reduce the temperature sensitivity of silica (expansion coefficient $\alpha =0.55\cdot 10^{-6}$ 1/K, thermo-optical coefficient $\beta =12.9\cdot 10^{-6}$ 1/K [18]) microspheres, a thin layer of material with a negative thermo-optic coefficient, for example, PMMA ($\alpha =\left(70-77\right)\cdot 10^{-6}$ 1/K, $\beta =-1\cdot 10^{-4}$ 1/K), can be applied [20,23]. For that purpose, some of the fabricated silica microspheres were dip-coated in a 3% PMMA–anisole solution. Before further coating, each layer was dried for at least 2 min. No annealing was performed. We have not measured the physical thickness of PMMA layer, as dip-coating and drying of a curved surface differs from a flat surface coating needed for ellipsometry. The temperature scan was performed in air to gauge the influence of the PMMA layer on the temperature sensitivity.

### 2.2. Experimental Arrangements

A basic scheme of the measurement system is shown in Figure 1b,c. The measurement system was set up using a 780 nm external cavity diode laser (ECDL, laser diode Thorlabs L785P090, spectral line < 1 MHz) to excite the resonances, an isolator (60 dB), a couple of mirrors, a focusing lens (F=1 cm), a Gadolinium Gallium Garnet (GGG) coupling prism to provide a light coupling into the WGMR, and a small Teflon tub to contain the liquid. A photo-detector (Photodiode Thorlabs PDA-36A) connected with an oscilloscope (Rigol DS1074 4 channel) was applied for signal analysis. The size of the WGMR microspheres was around (510 ± 50) μm. Additionally, a Peltier element (Melcor 2 × 2 cm), a temperature sensor (AD590), and a temperature controller (Thorlabs TTC001 or Thorlabs TED200C) were used to control the temperature. ECDL allowed a tunability of the central wavelength in the range of 10 nm.

**Figure 2:**
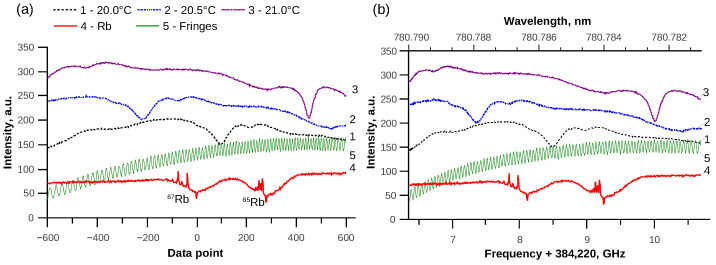
The temperature scanned WGMR resonance spectra for three different temperatures and two reference signals. (**a**) Before calibration, the laser scanning was obviously nonlinear, since at the beginning of the triangle signal the laser frequency scan was slower. (**b**) After calibration using both references for an almost linear absolute frequency scale.

WGMR was coupled to a prism, as described in reference [24]. While sweeping the laser frequency by changing the grating angle/position, transmittance spectra spanning about 3–4 GHz were recorded. Two reference signals were used for calibration: Rb saturation spectroscopy signal and interferometer fringes from the optical fiber, which has flat ends. The laser was adjusted in a mode to measure both ${}^{87}$Rb D2 and ${}^{85}$Rb D2 lines in the scanning region (see Figure 2a, curve 4). The hyperfine structure consisted of 3 Rb lines with known frequencies and 3 cross-over peaks for each isotope D2 line. The first reference was used to calculate the interferometer period of 48.78 MHz for the second reference, to follow the laser frequency drift due to change of ambient room conditions, and to indicate the absolute frequency. While observing the reference signal obtained from fringes, we deduced that the laser frequency scanning was nonlinear (see Figure 2a, curve 5), and additional calibration of the laser scan frequency was necessary (see Figure 2b). The second reference was used to both monitor the laser scan mode and linearize the frequency scanning.

### 2.3. Temperature Scanning

In a simplified case, the WGMR resonance shift df depends only on the termo-optical coefficient β and the expansion coefficient α. This dependence is linear from temperature *T* [25]:

(1) $$\begin{array}{c} \frac{df}{f}=-\frac{d\lambda }{\lambda }=-\left(\frac{1}{R}\frac{\partial R}{\partial T}+\frac{1}{n_{R}}\frac{\partial n_{R}}{\partial T}\right)dT=-\left(\alpha +\beta \right)dT, \end{array}$$

where λ is the wavelength of the laser, $n_{R}$ is the refractive index, and *R* is the radius of the sphere. This equation has been used by Guan et al. [26] to link the temperature with the WGMR resonance shift using a similar measurement setup in both air and water.

The temperature scan was performed by a Peltier element. A heating-cooling cycle from 20 ${}^{{}^{\circ}}$C to 25 ${}^{{}^{\circ}}$C with a step of 0.5 ${}^{{}^{\circ}}$C (see Figure 2b, curves 1, 2, 3) was performed. The temperature was changed using the temperature controller, and after achieving the temperature stability after at least 2 min, the transmission spectra with references were recorded for each step. Additionally, a temperature gradient was observed since the temperature sensor was located at the base of the Peltier element under the prism, and the resonator was close to the top of the prism (see Figure 1c). Temperature calibration was performed, and for every variation by 1 ${}^{{}^{\circ}}$C measured by this temperature sensor we observed a variation of 0.71 ${}^{{}^{\circ}}$C measured by WGMR sensor. Therefore, when calculating the actual WGMR resonance shift for 1 ${}^{{}^{\circ}}$C and the sum of coefficients, the gradient had to be taken into consideration. WGMR resonance spectra were recorded by the scan method after the temperature was stabilized. The measurements were performed in 3 different environments: air, water, and 5% glucose water solution.

### 2.4. Mathematical Modeling

Computer modeling was done using finite element method (FEM) simulations with *COMSOL Multiphysics* software. Simulations were done in 2D axisymmetric mode with the Wave Optics module using Electromagnetic Waves, Frequency Domain physics. The model consisted of a spherical 2R = 550 μm silica resonator, a PMMA layer of different thicknesses, and an air domain surrounding the resonator. The refractive indices, size of the resonator, and thickness of the coating layer were given parametrically as the functions of temperature, thermo-optic coefficient, and thermal expansion coefficient. The main goal was to find the frequency of the fundamental mode with a certain azimuthal mode number and find the frequency of the same mode for a different temperature, thereby determining the temperature-dependent frequency shift.

## 3. Results

### 3.1. Temperature Measurements in Air and Liquid

**Figure 3:**
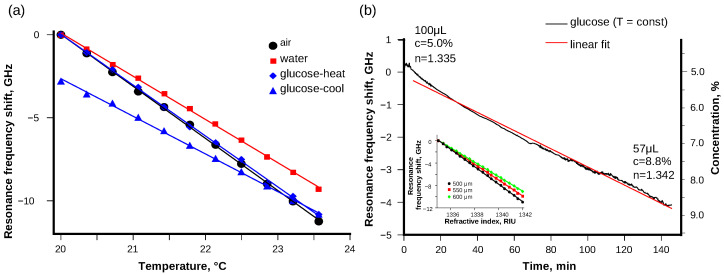
The frequency shifts depending on temperature for WGMR (**a**) in air, water, and 5% glucose solution are linear and can be fitted with linear approximations; however, for glucose, the frequency shift is different when heating or cooling. Therefore, it consists of two separate parts: (**b**) the independent shift of resonance spectra for a 5% glucose solution in stable temperature, and the drop evaporates and the glucose concentration increases, which changes the refractive index. Inset shows the modeling results for the resonance frequency shift depending on the refractive index of the surrounding media for 500, 550, and 600 μm microspheres.

Using the individual spectra recorded with the scan method for each temperature, the shift of resonance frequency from the temperature was calculated. Using the Rb saturation spectroscopy signal as a reference, it was possible to eliminate laser drift from the temperature-induced WGMR resonance shift. The sensor response to temperature was indeed linear, which had a good correlation with theoretical assumptions and previously published results [1,4,15,27]. The frequency shift in air was (−3030 ± 20) MHz/K (Figure 3a, black dots). In the water drop the frequency shift was slower than in air with (−2621 ± 24) MHz/K (Figure 3a, red squares). In a drop of 5% glucose solution, different results were observed depending on the temperature scan direction. The linear shift of the frequency for the temperature interval of 20–25 ${}^{{}^{\circ}}$C when heating was (−3064 ± 31) MHz/K (Figure 3a, blue diamonds), and for the interval of 25 ${}^{{}^{\circ}}$C–20 ${}^{{}^{\circ}}$C when cooling it was (−2267 ± 27) MHz/K (Figure 3a, blue triangles). The results suggest the presence of an additional resonance shift mechanism in the glucose solution drop.

Shifts in the WGMR resonance frequency can be caused not only by changes in size and refractive index of the resonator material due to changes in temperature but also by the variations in the surrounding medium refractive index [11]:

(2) $$\begin{array}{c} \frac{\Delta f}{f}=\frac{\Delta n_{m}}{n_{m}}F+\frac{\Delta n_{R}}{n_{R}}\left(1-F\right)+\frac{\Delta R}{R}, \end{array}$$

where $n_{m}$ is the refractive index of the surrounding medium, and *F* is a sensitivity function. The drop of liquid was relatively small and evaporated as the experiment progressed. As a result, the concentration of the glucose solution increased over time. The refractive index of a glucose solution depends on the concentration [28] and could contribute to an additional WGMR resonance shift [29].

To confirm the influence of resonance shift on the surrounding medium, the WGMR resonance was observed in a glucose solution drop at a constant temperature. The WGMR resonances shifted over time (Figure 3b). Initially, the volume of the drop was about 100 μL. As the water evaporated, the WGMR resonance shifted linearly. After an hour, when a significant part of the drop had evaporated, only a thin layer of liquid covered the WGMR. This made the observed WGM resonance position less stable and could be seen as increased noise of the WGMR resonance position. Finally, (57 ± 2) μL of the glucose solution was left. The left over liquid volume was evaluated using automatic micro-liter pipette (10–100 μL). The volume of the drop multiplied by the concentration is constant, which means the glucose concentration reached 8.8%. The refractive index of the glucose solution increased from 1.335 to 1.342 [30], and the WGM resonance shifted by −4001 MHz. Limit of detection was 12 MHz, and allowed us to detect a 0.01% change in glucose concentration or $1.5\cdot 10^{-5}$ RIU. Modeling results shown in Figure 3b inset confirm the WGM resonance shift induced by the surrounding media. Increasing the microsphere size decreased the sensitivity to the surrounding media. These results justify the different temperature dependence for a 5% glucose solution during heating and cooling cycles as the temperature scan in each direction took about 30 min.

Using the dependence of frequency shift on the temperature, the sum of thermo-optical and expansion coefficients can be found from Equation (1). The sum of coefficients obtained during the experiment is presented in Table 1. The calculated sum in liquid was lower than in air, which suggests the temperature gradient in the former case is different than in air because of both the larger heat capacity and the evaporation of liquid. Previously, the value α+β has been reported to be $9.23\cdot 10^{-6}$ 1/K at about 1531 nm for 430 μm silica microsphere [1].

### 3.2. Temperature Dependence in the Case of PMMA Coating

**Figure 4:**
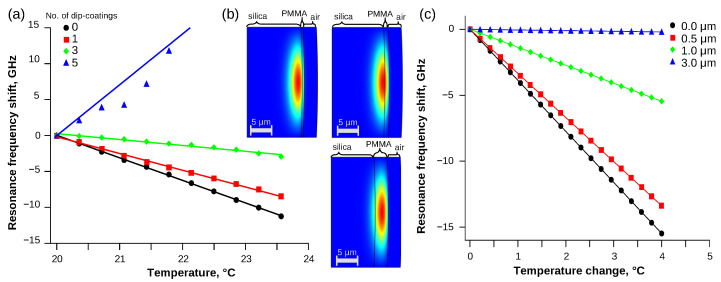
Experimental results show the frequency shift depending on temperature for WGMR in the air (**a**) coated with the PMMA layer is linear and can be fitted with linear approximation. The temperature sensitivity decreased as more layers were added until reversing the resonance shift direction for thicker PMMA layers. Mathematical modeling shows (**b**) fundamental mode moving inside the PMMA coating layer when the thickness increased from 0.5 to 1.0 and 3.0 μm. (**c**) The temperature sensitivity of the coated WGMR decreased as the PMMA thickness on the 550 μm silica microsphere increased.

The temperature sensitivity of the silica microsphere can be modified using thin coatings. The WGMR resonances shifted differently for the WGMRs dip-coated with PMMA (see Figure 4a and Table 2). As the microspheres were dipped multiple times, it was assumed the PMMA layer thickness increased. The resonances of the microspheres dipped once in PMMA solution were shifted by (−2370 ± 180) MHz/K (Figure 4a, red squares), slightly less than samples without any PMMA. After dipping the microsphere three times, the resonances shifted (−1070 ± 400) MHz/K, which was approximately three times less than without the PMMA layer (Figure 4a, green diamonds). The resonance of WGMR dipped five times was shifted by (6800 ± 1900) MHz/K (Figure 4a, blue triangles), which is at least double that without PMMA. Furthermore, the resonance shift direction was reversed. The PMMA layer thickness depends on many different coating parameters: solution concentration, the solvent used, solution temperature, dip time, dipping and towing speed in and out of the solution, etc.

The modeling results are shown in Figure 4b,c and Table 2. When the thickness of PMMA coating was increased, the electromagnetic field concentrated in the fundamental mode shifted from being located in silica resonator (0.5 μm coating) to being in the PMMA coating layer (3.0 μm coating). Similarly to experimental results, the temperature sensitivity decreased with increasing the thickness (Figure 4c).

## 4. Discussion

The temperature scanning was linear in air and the water drop. Using silica microsphere WGMR, a 3.3 mK temperature change could be detected in air and 3.8 mK in liquid. The experimentally observed resonance shift in air (−3030 ± 20 MHz/K) was smaller than the modeling of silica without any PMMA coating (−3870 MHz/K). One can assume that there is an additional temperature gradient between prism and WGMR. The rate of heat conduction does depend on the area. The area of contact between the microspheres and prism is small compared to the rest of the WGMR surface surrounded by air. When the temperature was varied by 1 ${}^{{}^{\circ}}$C, the temperature of WGMR changed only by about 0.54 ${}^{{}^{\circ}}$C, as the thermal stability was achieved. In the case of liquid drop surrounding the WGMR, thermal stability was achieved even “faster,” when the WGMR temperature changed only by 0.47–0.48 ${}^{{}^{\circ}}$C.

The analysis of results, obtained in the 5% glucose solution, were rather complex, and require further investigations because the resonance shift caused by temperature scanning varied during heating or cooling. As any possible laser frequency drift was eliminated by using the Rb saturation signal as the reference, the additional resonance frequency shift was linked with the change in refractive index due to water evaporating from the drop in the glucose solution. The evanescent field around the WGMR appeared to extend the size, making the WGMR very sensitive to the surrounding media and temperature. Similar temperature sensitivity to the media has been observed by Kim et al. [19] and used to achieve thermal stability. The sensitivity to the refractive index of the glucose solution drop is shown in Figure 3b. Our measurement system is very sensitive to the refractive index of the surrounding medium. Using silica microsphere WGMR, $1.5\cdot 10^{-5}$ RIU change could be detected. This effect can be used to measure the variations in the low concentrations of solutions.

When the temperature is scanned, the change of the refractive index of the media should be taken into consideration. When the index of refractive of the solution increases, as the water in drop evaporates, the WGMR resonance frequency decreases. Similarly, the refractive index of the water and glucose solution depends on the temperature of the liquid [30]. As the temperature increases, the refractive index of the liquid decreases and the resonance frequency should increase. As a result, two effects of the resonance shift due to WGMR and resonance shift due to the surrounding medium fight compete with each other. This effect should be investigated further.

The thermal sensitivity can also be influenced by coating of WGMR with a thin layer of PMMA. As the PMMA thickness increased, the sum of α and β decreased and eventually changed the sign. Many aspects influence the thickness and structure of PMMA coating, which require further investigation. Similar results have been reported by Li et al. [22] for polydimethylsiloxane (PDMS) coated on silica toroidal microresonators. Results reported by Jestin et al. [23] also show reduced temperature tuning in larger silica microspheres, coated with an estimated 0.55 μm PMMA layer. Mathematical modeling confirmed the trend of temperature sensitivity decreasing for thicker coatings. Such silica microspheres and thin PMMA or a similar coating combination could be useful to reduce temperature sensitivity and simultaneously increase sensitivity, for example, to humidity, which is added by the PMMA layer [5]. The optimal thickness of PMMA to compensate temperature sensitivity for 550 μm microsphere was determined to be about 3 μm.

## 5. Conclusions

In conclusion, we have demonstrated the applicability of temperature measurements using the WGMR in both air and liquid environments. We have also shown that the WGMR allows the measurements of the refractive index variations in the evaporating glucose–water solution droplet. We have shown that the thermal tuning of WGMR can be reduced by coating WGMRs with a thin layer of PMMA. Dip-coating the silica microsphere multiple times significantly reduced the resonance shift, partially compensating for the positive thermo-optical coefficient of silica.

In the future, the proposed temperature scanning method could be used for measurements if no applicable WGMR resonances are detected at room temperature in the laser scan region. The system can also be used to detect minor refractive index changes in surrounding liquid media. Temperature sensitivity can be manipulated using thin coatings to potentially decrease unwanted dependencies and add or increase additional sensitivities.

## Figures and Tables

**Table 1:** Measured WGMR resonance shifts from temperature and the sum of α and β coefficients in air and water and glucose solution drops.

Environment	df/dT	α+β
	(MHz/K)	$\left(10^{-6}K^{-1}\right)$
air	−3030 ± 20	7.88 ± 0.05
water	−2621 ± 24	6.81 ± 0.06
5% glucose solution	−2666 ± 58	6.93 ± 0.15

**Table 2:** (left panel) The measured temperature-dependent resonance shifts and the sums of α and β coefficients for PMMA-coated microspheres in air. (right panel) Modeling results for the 0, 0.5, 1.0, and 3.0 μm thick PMMA coatings on 550 μm silica microspheres.

No. of	df/dT	α+β	Thickness	df/dT
Dips	(MHz/K)	$\left(10^{-6}K^{-1}\right)$	(μm)	(MHz/K)
ine 0	−3030 ± 20	7.88 ± 0.05	0	−3870
ine 1	−2370 ± 180	6.2 ± 0.5	0.5	−3345
ine 3	−1070 ± 400	2.8 ± 1.0	1.0	−1364
ine 5	6800 ± 1900	−17.6 ± 4.9	3.0	−49

## Data Availability

The data presented in this study are available on request from the corresponding author.

## Abbreviations

The following abbreviations are used in this manuscript:

WGMR	Whispering Gallery Mode Resonator
Q	Quality factor
PMMA	PolyMethyl MethAcrylate
PDMS	PolyDiMethylSiloxane
GGG	Gadolinium Gallium Garnet
ECDL	External Cavity Diode Laser
SMF	Single Mode Fiber
FEM	Finite Element Method
RIU	Refractive Index Unit

## References

[1] Ma Q., Rossmann T., Guo Z. (2008). Temperature sensitivity of silica micro-resonators. J. Phys. D. Appl. Phys..

[2] Rahman A. (2011). Temperature sensor based on dielectric optical microresonator. Opt. Fiber Technol..

[3] Ward J.M., Dhasmana N., Nic Chormaic S. (2014). Hollow core, whispering gallery resonator sensors. Eur. Phys. J. Spec. Top..

[4] He C., Sun H., Mo J., Yang C., Feng G., Zhou H., Zhou S. (2018). Temperature sensor based on high-Q polymethylmethacrylate optical microbubble. Laser Phys..

[5] Petermann A., Hildebrandt T., Morgner U., Roth B., Meinhardt-Wollweber M. (2018). Polymer Based Whispering Gallery Mode Humidity Sensor. Sensors.

[6] Reinis P.K., Milgrave L., Draguns K., Brice I., Alnis J., Atvars A. (2021). High-Sensitivity Whispering Gallery Mode Humidity Sensor Based on Glycerol Microdroplet Volumetric Expansion. Sensors.

[7] Li Y.L., Barker P.F. (2018). Characterization and Testing of a Micro-g Whispering Gallery Mode Optomechanical Accelerometer. J. Light. Technol..

[8] Armani A.M., Kulkarni R.P., Fraser S.E., Flagan R.C., Vahala K.J. (2007). Label-Free, Single-Molecule Detection with Optical Microcavities. Science.

[9] Righini G., Soria S. (2016). Biosensing by WGM Microspherical Resonators. Sensors.

[10] Brice I., Grundsteins K., Atvars A., Alnis J., Viter R., Ramanavicius A. (2020). Whispering gallery mode resonator and glucose oxidase based glucose biosensor. Sens. Actuators B Chem..

[11] Foreman M.R., Swaim J.D., Vollmer F. (2015). Whispering gallery mode sensors. Adv. Opt. Photonics.

[12] Llopis O., Merrer P.H., Bouchier A., Saleh K., Cibiel G. (2010). High-Q optical resonators: Characterization and application to stabilization of lasers and high spectral purity microwave oscillators. Proc. SPIE.

[13] Gorodetsky M.L., Savchenkov A.A., Ilchenko V.S. (1996). Ultimate Q of optical microsphere resonators. Opt. Lett..

[14] Spillane S.M., Kippenberg T.J., Painter O.J., Vahala K.J. (2003). Ideality in a Fiber-Taper-Coupled Microresonator System for Application to Cavity Quantum Electrodynamics. Phys. Rev. Lett..

[15] Kavungal V., Farrell G., Wu Q., Mallik A.K., Semenova Y. (2018). Thermo-optic tuning of a packaged whispering gallery mode resonator filled with nematic liquid crystal. Opt. Express.

[16] Kobatake T., Kato T., Itobe H., Nakagawa Y., Tanabe T. (2016). Thermal Effects on Kerr Comb Generation in a CaF_2_ Whispering-Gallery Mode Microcavity. IEEE Photonics J..

[17] Jiang X., Yang L. (2020). Optothermal dynamics in whispering-gallery microresonators. Light. Sci. Appl..

[18] Toyoda T., Yabe M. (1983). The temperature dependence of the refractive indices of fused silica and crystal quartz. J. Phys. D. Appl. Phys..

[19] Kim E., Foreman M.R., Baaske M.D., Vollmer F. (2015). Thermal characterisation of (bio)polymers with a temperature-stabilised whispering gallery mode microsensor. Appl. Phys. Lett..

[20] Han M., Wang A. (2007). Temperature compensation of optical microresonators using a surface layer with negative thermo-optic coefficient. Opt. Lett..

[21] Savchenkov A., Matsko A. (2018). Calcium fluoride whispering gallery mode optical resonator with reduced thermal sensitivity. J. Opt..

[22] Li B.B., Wang Q.Y., Xiao Y.F., Jiang X.F., Li Y., Xiao L., Gong Q. (2010). On chip, high-sensitivity thermal sensor based on high-Q polydimethylsiloxane-coated microresonator. Appl. Phys. Lett..

[23] Jestin Y., Berneschi S., Conti G.N., Chiappini A., Ferrari M., Righini G.C. Micro resonator stabilization by thin film coating. Proceedings of the ICTON 2009: 11th International Conference on Transparent Optical Networks.

[24] Alnis J., Brice I., Pirktina A., Ubele A., Grundsteins K., Atvars A., Viter R. (2017). Development of optical WGM resonators for biosensors. Proc. SPIE.

[25] Atvars A. (2021). Analytical description of resonances in Fabry–Perot and whispering gallery mode resonators. J. Opt. Soc. Am. B.

[26] Guan G., Arnold S., Otugen M.V. (2006). Temperature Measurements Using a Microoptical Sensor Based on Whispering Gallery Modes. AIAA J..

[27] Yang Y., Wang M., Shen Y., Meng L., Zhang L., Xu W., Wang K.W. (2019). Experimental demonstration of laser-machined high-Q microrod resonator for thermal sensing. Proc. SPIE.

[28] Malinin A.V., Zanishevskaja A.A., Tuchin V.V., Skibina Y.S., Silokhin I.Y. (2012). Photonic crystal fibers for food quality analysis. Proc. SPIE.

[29] Miri N., Mohammadzaheri M. (2014). Optical sensing using microspheres with different size and material. IEEE Sens. J..

[30] Belay A., Assefa G. (2018). Concentration, Wavelength and Temperature Dependent Refractive Index of Sugar Solutions and Methods of Determination Contents of Sugar in Soft Drink Beverages using Laser Lights. J. Lasers Opt. Photonics.

